# Arginase: The Emerging Therapeutic Target for Vascular Oxidative Stress and Inflammation

**DOI:** 10.3389/fimmu.2013.00149

**Published:** 2013-06-12

**Authors:** Zhihong Yang, Xiu-Fen Ming

**Affiliations:** ^1^Vascular Biology, Division of Physiology, Department of Medicine, University of Fribourg, Fribourg, Switzerland

**Keywords:** arginase, eNOS, superoxide, adhesion molecules, signal transduction pathway

## Abstract

Oxidative stress and inflammation in the vascular wall are essential mechanisms of atherosclerosis and vascular dysfunctions associated with risk factors such as metabolic diseases, aging, hypertension, etc. Evidence has been provided that activation of the vascular endothelial cells in the presence of the risk factors promotes oxidative stress and vascular inflammatory responses, leading to acceleration of atherosclerotic vascular disease. Increasing number of studies from recent years demonstrates that uncoupling of endothelial nitric oxide synthase (eNOS), whereby the enzyme eNOS produces detrimental amount of superoxide anion ${\text{O}}_{2}^{-}$ instead the vasoprotective nitric oxide (NO^⋅^), plays a critical role in vascular dysfunction under various pathophysiological conditions and in aging. The mechanisms of eNOS-uncoupling seem multiple and complex. Recent research provides emerging evidence supporting an essential role of increased activity of arginases including arginase-I and arginase-II in causing eNOS-uncoupling, which results in vascular oxidative stress and inflammatory responses, and ultimately leading to vascular diseases. This review article will summarize the most recent findings on the functional roles of arginases in vascular diseases and/or dysfunctions and the underlying mechanisms in relation to oxidative stress and inflammations. Moreover, regulatory mechanisms of arginases in the vasculature are reviewed and the future perspectives of targeting arginases as therapeutic options in vascular diseases are discussed.

## Introduction

Atherosclerotic cardiovascular disease and vascular complications associated with risk factors such as diabetes mellitus, hypercholesterolemia, hypertension, aging, etc., remain the most important challenge for our society (Sidney et al., 2013). Mechanisms of pathogenesis of atherosclerosis are complex interplay between bloodstream cells and arterial wall components that leads to a chronic state of vascular oxidative stress and inflammation (Hansson and Hermansson, 2011). In the past decades, unambiguous evidence has been provided that heightened oxidative stress and vascular wall inflammation are the key mechanisms for initiation and progression of atherosclerosis and vascular diseases associated with the risk factors (Hansson and Hermansson, 2011). Oxidative stress not only chemically modifies native LDL to the highly atherogenic oxidized LDL which is readily taken up by infiltrated macrophages in the intima of the vascular wall, resulting in foam cell formation, but also causes vascular cell damage that triggers inflammatory responses in the vascular wall and facilitates pathogenesis of vascular diseases, leading to rupture of lipid-rich vascular lesions, the life-threatening events, such as acute myocardial infarction and stroke (Faxon et al., 2004; Hansson and Hermansson, 2011). Therefore, elucidation of mechanisms underlying oxidative stress and inflammations in the vascular wall will have important impact in understanding atherosclerosis and vascular diseases associated with cardiovascular risk factors and will eventually lead to novel and effective therapeutic modalities.

## Oxidative Stress, Inflammation, and Vascular Disease

Oxidative stress is characterized with the excessive production of oxidant molecules that overwhelm the anti-oxidant defense systems, resulting in oxidative damage (Lonn et al., 2012). The oxidant molecules include radicals and non-radicals may cause damage of DNA, proteins, and lipids, leading to alterations in cellular functions or cell death (Lonn et al., 2012). Reactive oxygen species (ROS) such as superoxide anion (${\text{O}}_{2}^{-}$), hydrogen peroxide (H_2_O_2_) and nitric oxide (NO^⋅^) are important signaling molecules involved in the regulation of vascular functions, including vascular relaxations, inflammatory responses, and cell proliferation (Sundaresan et al., 1995; Yang and Ming, 2006b; Murphy et al., 2011). Under physiological conditions, the production of these molecules is spatially and temporally regulated, participating in the maintenance of homeostasis of vascular functions (Sundaresan et al., 1995; Yang and Ming, 2006b). Multiple enzymes involved in oxidative stress within the vascular wall can be stimulated or up-regulated in the presence of cardiovascular risk factors, leading to excessive production of ROS and cellular damage (Lonn et al., 2012). ${\text{O}}_{2}^{-}$ is the parent ROS molecule produced by the one electron reduction of oxygen catabolized by various enzymes including NADPH oxidase, cyclooxygenase, lipoxygenases, cytochrome P450 enzymes, enzymes in the mitochondrial electron transport chain (Yang and Lüscher, 2002), and also endothelial NO^⋅^ Synthase (eNOS, see below). ${\text{O}}_{2}^{-}$ is then dismuted by superoxide dismutase (SOD) to H_2_O_2_ which is either detoxified to H_2_O by peroxiredoxins, glutathione peroxidases, and catalase or metabolized to the powerful oxidant molecules such as hydroxyl radical (OH^⋅^), peroxynitrite (${\text{ONOO}}_{\cdot }^{-}$), and hypochlorous acid (HOCl) through enzymatic or non-enzymatic reactions. For detailed description on ROS generation and reaction as well as the role of oxidative stress in pathogenesis of atherosclerosis, please refer to the review article (Lonn et al., 2012).

Chronic vascular inflammation is the fundamental mechanism of vascular diseases associated with variety of risk factors, contributing to pathogenesis of atherosclerosis and plaque rupture, leading to acute coronary syndromes (Hansson and Hermansson, 2011). Macrophages, T cells and other immune cells, pro-inflammatory cytokines are found in the atherosclerotic lesions. Innate as well as adaptive immune responses are identified in atherosclerosis (Hansson and Hermansson, 2011). At the cellular and molecular levels, oxidative stress, vascular inflammation, as well as endothelial cell dysfunction which is mainly reflected by decreased vasoprotective endothelial NO^⋅^ bioavailability intertwine with each other, represent the major mechanisms leading to exaggerated atherosclerosis in the presence of risk factors (Lonn et al., 2012). Because of the complex interaction among these events, it is not easy to delineate their causal relationship in the pathogenesis of vascular diseases. Under physiological conditions, in the absence of risk factors, the endothelial cells express negligible levels of adhesion molecules such as ICAM-1 and VCAM-1 for inflammatory cells and low levels of the coagulation enzyme tissue factor (Viswambharan et al., 2004; Ming et al., 2009, 2010), whereas in the presence of the risk factors, these molecules are up-regulated in the cells, which may enhance monocyte–endothelial cell interaction and activation of coagulation cascade, participating in the initiation and progression of atherosclerotic plaque formation and thrombus formation (Camici et al., 2006). The role of inflammation and underlying mechanisms in atherogenesis and atherothrombosis are comprehensively reviewed by many articles (Faxon et al., 2004; Hansson and Hermansson, 2011; Lonn et al., 2012). In this review article, we will mainly discuss the role and mechanisms of the enzyme *arginase* in vascular endothelial dysfunction, oxidative stress, and inflammation in the pathogenesis of vascular diseases.

## Endothelial Dysfunction and eNOS-Uncoupling

**Figure 1:**
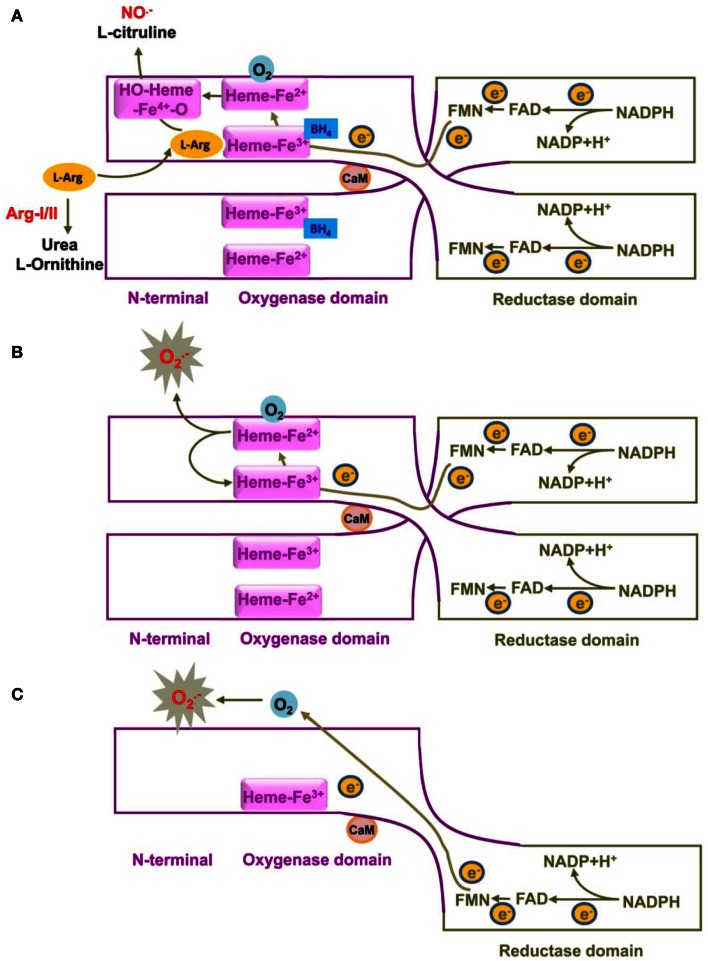
**Schematic illustration of mechanisms of eNOS in catalyzing NO^⋅^or ${\text{O}}_{2}^{•-}$ production**. eNOS monomer consists of an N-terminal heme-containing oxygenase domain, a C-terminal flavin-containing reductase domain and a regulatory CaM-binding linker sequence. Monomer can bind to CaM, but not co-factor BH_4_ or substrate L-arginine. **(A)** A functional eNOS is a homodimer and transfers electron from NADPH from the reductase domain of one monomer, via FAD and FMN, to the heme in the oxygenase domain of the other monomer, where BH_4_, oxygen, and L-arginine are bound. At the heme site, the reduction of Fe^3+^ to Fe^2+^ facilitates oxygen binding to the heme group to form a transient Fe^4+^-O_2_ complex that is further reduced to form a hydroxylating heme-Fe^4+^-oxo species, which in turn oxidizes L-arginine to NO^⋅^ and L-citrulline. Due to the nature of the electron transfer in trans, only eNOS dimer, but not the monomer, is functional in catalyzing NO^⋅^ production. The binding of CaM to eNOS, upon an increased intracellular Ca^2+^ concentration in response to agonist stimulation, facilitates the electron transfer from NADPH to both flavins (FAD and FMN) as well as to the heme and ultimately the NO^⋅^ production. **(B,C)** Under pathological conditions that cause BH_4_ deficiency or L-arginine depletion, “eNOS-uncoupling” occurs – that is, uncoupling of NADPH oxidation and NO^⋅^ synthesis, with oxygen instead of L-arginine as terminal electron acceptor, resulting in the formation of ${\text{O}}_{2}^{-}$ instead of NO^⋅^ from eNOS. eNOS-derived ${\text{O}}_{2}^{-}$ production mainly comes from uncoupled eNOS dimer **(B)**, whereas monomer has only a limited capacity to reduce molecular oxygen to ${\text{O}}_{2}^{-}$ **(C)**. For simplicity and clarity, the flow of electrons in trans is only shown from one monomer to the other monomer. The diagram is not to scale and is made based on these publications (Griffith and Stuehr, 1995; Abu-Soud et al., 1997; Vasquez-Vivar et al., 1998). CaM, calmodulin; BH4, tetrahydrobiopterin; NADPH, nicotinamide adenine dinucleotide phosphate; FAD, flavin adenine dinucleotide; FAM, flavin mononucleotide.

The endothelium regulates vascular functions by multiple mechanisms (Yang and Lüscher, 2002). It is well established that the decreased bioavailability of the vasoprotective endothelial NO^⋅^ molecule best reflects dysfunctional endothelium or endothelial dysfunction under pathological conditions and in the presence of risk factors (Forstermann and Sessa, 2012). It represents one of the most important early markers and mechanisms of cardiovascular disease and also predicts the future atherosclerotic disease progression (Schachinger et al., 2000). The endothelial NO^⋅^ is produced by the eNOS from the semi-essential amino acid l-arginine in the presence of oxygen and co-factors such as reduced nicotinamide adenine dinucleotide phosphate (NADPH), flavin adenine dinucleotide (FAD), flavin mononucleotide (FMN), and tetrahydrobiopterin (BH_4_). Electrons from NADPH are transferred in trans from the carboxyl terminal reductase domain of one eNOS monomer, via the flavins FAD and FMN, to the heme in the amino-terminal oxygenase domain of the other monomer, where BH_4_, oxygen, and l-arginine are bound (Figure 1). At the heme site, the electrons activate O_2_, so that l-arginine is oxidized to l-citrulline and NO^⋅^. Due to the nature of the electron transfer in trans, only eNOS dimer, but not the monomer, is functional in catalyzing NO production. This process, especially the electron transfer from FMN to heme is facilitated by calmodulin binding to eNOS. An increase in intracellular Ca^2+^ concentration in the endothelial cells upon agonist stimulation enhances calmodulin binding affinity to eNOS, promoting flow of electron transfer and NO^⋅^ production (Forstermann and Sessa, 2012). In addition to intracellular Ca^2+^ concentration, eNOS also requires co-factor BH_4_ for enzyme activity. Deficiency in BH_4_ or inactivation of BH_4_ by oxidative stress has been show to destabilize eNOS dimer and decreases NO^⋅^ production (Crabtree and Channon, 2011). Interestingly, under this condition “eNOS-uncoupling” may occur – that is, uncoupling of NADPH oxidation and NO^⋅^ synthesis, with oxygen instead of l-arginine as terminal electron acceptor, resulting in the formation of ${\text{O}}_{\cdot }^{-}$ instead of NO^⋅^ from eNOS (Forstermann and Sessa, 2012) (Figure 1). Evidence has been shown that eNOS-uncoupling plays an important part in endothelial dysfunction in many diseases including atherosclerosis, hypertension, myocardial ischemia/reperfusion injury, diabetes mellitus, as well as aging (please refer to the review article by Kietadisorn et al. (2012). The concept to improve endothelial function under these conditions have been evolved from increasing eNOS gene expression to restoring or recoupling eNOS function, since eNOS gene expression are not decreased and even enhanced in the majority of the conditions. For example, eNOS expression in atherosclerotic arteries and arteries from diabetes mellitus as well as in arteries from aged animals is usually compensatorily increased or not changed (Cosentino et al., 1997; van der Loo et al., 2000; d’Uscio et al., 2001; Ming et al., 2004; Desrois et al., 2010; Rajapakse et al., 2011). Hence, elucidation of mechanisms of eNOS-uncoupling becomes essential for future therapeutic intervention to improve endothelial function in the clinical settings.

The mechanism of eNOS-uncoupling seems multiple and includes oxidation of the co-factor BH_4_, decreased intracellular availability of the substrate l-arginine either due to increased arginase activity or accumulation of endogenous methylarginines such as asymmetric dimethyl-l-arginine (ADMA) that competes with l-arginine for eNOS binding (Forstermann and Sessa, 2012). Moreover, S-glutathionylation of eNOS has been proposed as yet another mechanism of eNOS-uncoupling (Chen et al., 2010). In this review article, we will focus on the roles of arginase in eNOS dysfunction.

## Arginase Promotes eNOS-Uncoupling, Oxidative Stress, and Inflammation

In humans and mammals, there are two isoforms of arginases: arginase-1 (Arg-I) and arginase-II (Arg-II), which is encoded by two separate genes. The human Arg-I gene which maps to chromosome 6q23, encodes a 322 amino acid protein (Dizikes et al., 1986a,b; Sparkes et al., 1986). The human Arg-II gene which maps to chromosome 14q24.1, encodes a 354 amino acid protein (Gotoh et al., 1996, 1997; Vockley et al., 1996) The two enzymes have similar structural properties, enzyme characteristics and share more than 50% of homology of their amino acid residues, with 100% homology in those areas critical to enzymatic function (Gotoh et al., 1996; Morris Jr. et al., 1997; Vockley et al., 1996). Arg-I is a cytosolic enzyme abundantly expressed in liver (Haraguchi et al., 1987). It hydrolyzes l-arginine to urea and l-ornithine, is the sixth and final enzyme of the hepatic urea cycle responsible for elimination of excessive nitrogen generated primarily by the metabolism of amino acids which are derived from the food intake or from endogenous protein catabolism (Crombez and Cederbaum, 2005). Arg-I knockout mice exhibited severe symptoms of hyperammonemia, and died between postnatal days 10 and 14 (Iyer et al., 2002). Arg-I deficiency due to gene mutation has been identified and characterized in humans. These patients reveal urea cycle disorder, hyperargininemia and exhibit neurologically based clinical symptoms in early childhood, including progressive neurologic impairment, development retardation, and hepatic dysfunction associated with cirrhosis and carcinoma (Crombez and Cederbaum, 2005; Tsang et al., 2012). Although this enzyme is largely confined to the liver, it is also present in many extrahepatic tissues such as stomach, pancreas, and lung (Choi et al., 2012). Arg-I gene expression is inducible by a variety of stimuli. Upregulation of Arg-I has been reported in macrophages upon stimulation by cAMP, IL-4, and TGF-β (Morris, 2000) and Arg-I expression is increased in aging vasculature of rats (White et al., 2006). Unlike Arg-I, Arg-II is a mitochondrial enzyme and most abundantly expressed in kidney and widely expressed in many extrahepatic tissues such as brain, prostate, intestine, and pancreas (Gotoh et al., 1996; Vockley et al., 1996; Choi et al., 2012) and is inducible in other organs and cells including macrophages and vascular endothelial cells (Ming et al., 2012; Yepuri et al., 2012). As compared to Arg-I, the function of Arg-II is not well characterized. Studies in the vascular endothelial cells suggest that these two isoforms share similar functions, i.e., metabolizing l-arginine to urea and l-ornithine, whereby enhanced Arg-I or/and Arg-II limits l-arginine bioavailability for NO^⋅^ production, leading to endothelial dysfunction (Xia et al., 1996; Kim et al., 2009).

This hypothesis, however, requires confirmation by further experimental evidence. Given that the concentration of l-arginine in adult human and mouse plasma (0.1 mmol/L) as well as intracellular l-arginine concentration (0.05–0.2 mmol/L) far exceed the *K*_m_ of eNOS (2–20 μmol/L) (Morris Jr., 2002), a real intracellular l-arginine depletion does not seem present. Yet acute l-arginine supplementation in patients and animals has been shown to enhance NO^⋅^ production and endothelium-dependent relaxations, despite sufficiently high concentrations of l-arginine in the extracellular space, a situation known as “arginine paradox” (Kurz and Harrison, 1997). This finding led to several hypotheses of “relative” intracellular l-arginine deficiency. One hypothesis proposes that there might be different intracellular l-arginine pools for NO^⋅^ production (Topal et al., 2006; Closs et al., 2000). While exogenous l-arginine seems channeled to eNOS to produce NO^⋅^, the putative intracellular l-arginine pool is not freely exchangeable with the extracellular l-arginine, it is however accessible to eNOS and arginase (Topal et al., 2006; Closs et al., 2000). This model could explain the “l-arginine paradox” and the observation that inhibition of arginase stimulates NO^⋅^ production and overexpression of Arg-I or -II suppresses NO^⋅^ production in the endothelial cells, which is associated with only a mild reduction in intracellular l-arginine concentration (11–25% decrease) even in the presence of high extracellular concentration of l-arginine (0.4 mmol/L) (Li et al., 2001). Yet it is highly speculative. Another explanation is a “relative” intracellular deficiency of l-arginine that could be resulted from the increased levels of ADMA, the endogenous eNOS inhibitor, which blocks intracellular l-arginine utility by eNOS to produce NO^⋅^ (Antoniades et al., 2009). It is assumable that an increase in arginase activity in the presence of ADMA in endothelial cells would further significant limit intracellular l-arginine bioavailability for eNOS to produce NO^⋅^, although the intracellular l-arginine concentration is only mildly decreased. If the hypothesis of the “relative l-arginine deficiency” is true, supplementation of l-arginine aiming to enhance endothelial NO^⋅^ production and to treat vascular disease may not work. Too much l-arginine may even cause harmful effects due to production of other undesired metabolites from l-arginine (Dioguardi, 2011). Indeed, a randomized, double-blinded, placebo-controlled study in patients with acute myocardial infarction, the VINTAGE MI study, demonstrates that 6 months oral l-arginine supplementation (3 g three times a day on top of standard postinfarction therapy) does not have any benefits on vascular stiffness and left ventricular ejection fraction, but increases mortality (Schulman et al., 2006). In line with this result, another clinical study in patients with peripheral artery disease, the NO-PAIN study, shows decreased NO^⋅^ production and shortened walking distance in patients receiving l-arginine supplementation as compared to the placebo group (Wilson et al., 2007). The impact of l-arginine supplementation, particularly chronic supplementation for treatment of cardiovascular diseases does not seem beneficial, it is rather detrimental and should not be recommended in the clinical settings.

The underlying mechanisms of the detrimental effects of chronic l-arginine supplementation in patients are not clear. Several hypotheses have been discussed. As aforementioned, too much l-arginine may lead to exaggerated production of undesired metabolites through arginase, such as l-proline and l-ornithine which is further metabolized to polyamines (Durante et al., 2001; Wei et al., 2001; Yang and Ming, 2006a). l-proline is an essential component for collagen synthesis and polyamines are important factors supporting vascular smooth muscle cell proliferation (Durante et al., 2001; Wei et al., 2001; Yang and Ming, 2006a). These effects of arginase-derived products may be involved in vascular intimal thickening and vascular stiffness associated with vascular injury and aging (Durante et al., 2001; Wei et al., 2001; Yang and Ming, 2006a; Marinova et al., 2008). The effects of the l-arginine metabolites through arginase in endothelial cells are not clear. Strong evidence shows that elevated arginase expression and/or activity in endothelial cells limit NO bioavailability through eNOS-uncoupling, leading to oxidative stress and vascular inflammatory responses (see discussion below).

**Figure 2:**
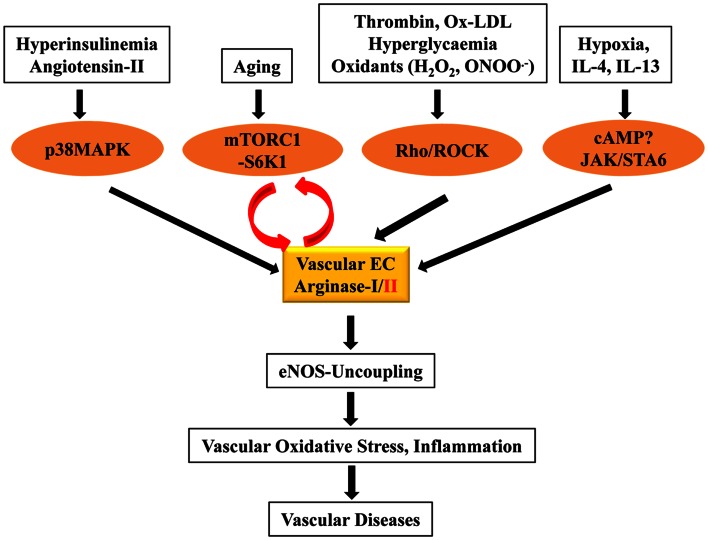
**The signaling mechanisms involved in upregulation of vascular arginase expression/activity in vascular endothelial cells (EC)**. Various cardiovascular risk factors such as hyperinsulinemia, aging, hyperglycemia, hypoxia, etc., upregulate Arg-I or/and Arg-II expression/activity through signaling pathways including p38MAPK, mTORC1-S6K, Rho/ROCK, and JAK/STAT6, leading to eNOS-uncoupling that ultimately causes vascular oxidative stress and inflammation contributing to the development of vascular diseases. Moreover, the mutual positive regulation between S6K1 and Arg-II gene expression accelerates oxidative stress and inflammation through eNOS-uncoupling.

The role of arginase including type-I and type-II isozyme in decreased endothelial NO^⋅^ production is well documented (please see review articles: Yang and Ming, 2006a; Vanhoutte, 2008). This effect of arginase has been demonstrated being the consequence of eNOS-uncoupling (Ming et al., 2004; Romero et al., 2008; Kim et al., 2009; Scalera et al., 2009; Shin et al., 2012; Yepuri et al., 2012). Since endothelial NO^⋅^ is an important anti-inflammatory molecule and suppresses expression of adhesion molecules such as VCAM-1, ICAM-1 (Lee et al., 2002), a positive association between plasma arginase level or peripheral blood mononuclear cell arginase level and soluble VCAM-1 and ICAM-1 is demonstrated in patients with sickle cell anemia and overweight subjects (Morris et al., 2005; Kim et al., 2012). In cultured human endothelial cells, genetic inhibition of Arg-II prevents ICAM-1 and VCAM-1 upregulation upon persistent insulin stimulation to mimic the hyperinsulinemia condition (Giri et al., 2012) and decreases their expression in senescent endothelial cells (Yepuri et al., 2012), demonstrating that Arg-II plays a role in endothelial inflammatory responses. This conclusion is further confirmed by the fact that overexpression of Arg-II gene in the non-senescent human endothelial cells enhances VCAM-1 and ICAM-1 levels (Yepuri et al., 2012). Importantly, our study further shows that in senescent human endothelial cells and aortas of old mice, the Arg-II (but not Arg-I) gene expression and activity is augmented and genetic silencing or ablation of Arg-II in senescent human cells or in old mice recouples eNOS function, leading to inhibition of oxidative stress and decrease in adhesion molecule expression *in vitro* cell culture and *in vivo* mouse aging models, resulting in decreased monocyte-endothelial interaction (Yepuri et al., 2012). Moreover, this study shows that inhibition of Arg-II gene is able to prevent or reverse endothelial senescence phenotype markers in the aging models (Yepuri et al., 2012), demonstrating the causal role of Arg-II in cardiovascular aging. The detrimental role of Arg-II in atherosclerotic vascular disease has also been recently evidenced in mice either with systemic deficiency of Arg-II (Ming et al., 2012) or in endothelial specific Arg-II transgenic mice (Vaisman et al., 2012). Enhanced endothelial arginases thus represent an important mechanism in inducing eNOS-uncoupling and the associated oxidative stress and inflammation in vasculature contributing to the development of vascular diseases (Figure 2). Elucidation of the regulatory mechanisms of arginases in the vasculature would provide rationales for the development of new drugs for treatment of cardiovascular disorders.

## Regulatory Mechanism of Arginases in Vascular Diseases

Studies investigating regulatory mechanisms of arginase gene expression and enzymatic activity so far are limited to Arg-I in murine macrophages (for details please refer to the most recently published review article by Pourcet and Pineda-Torra, 2013). There is little information available regarding the upstream regulatory mechanisms involved in gene expression and enzymatic activity of arginases in vascular cells. A few stimuli have been shown to upregulate arginase gene expression and/or enzymatic activity. It has been reported that transgenic expression of IL-13 in the lung of mouse, the T-helper type 2 cell effector cytokine, causes pulmonary arteriole remodeling and subsequently pulmonary hypertension, which is associated with enhanced expression of both Arg-I and Arg-II in the lung (Cho et al., 2013). Genetic deletion of Arg-II gene in this mouse partly prevents IL-13-induced pulmonary hypertensive phenotypes, suggesting the involvement of Arg-II (Cho et al., 2013). The study supports the notion of human studies showing that increased Arg-II expression in pulmonary endothelial cells is associated with pulmonary arterial hypertension (Xu et al., 2004). Whether inhibition of Arg-II reveals therapeutic effect in human pulmonary hypertension remains to be investigated and depends on the development of specific Arg-II inhibitors. In contrast to Arg-II, the role of Arg-I in this context is not known. The mechanisms of IL-13-induced Arg-II expression are not clear. In human pulmonary arterial smooth muscle cells, hypoxia is capable of inducing Arg-II expression, which is inhibited by cAMP (Chen et al., 2012). In contrast to this study, Wei et al. (2000) shows that cAMP, besides JAK/STAT6 upregulates Arg-I (not Arg-II) in rat aortic smooth muscle cells upon stimulation by IL-4 or IL-13. It is not clear whether this could be explained by the different biological properties of smooth muscle cells of different origins or species or the different stimuli used in their experimental settings.

Moreover, the GTPase RhoA and its down-stream kinase ROCK have been shown to upregulate arginase activity with or without augmentation of the corresponding gene expression of the isozymes. Oxidative stress, such as hydrogen peroxide (H_2_O_2_) and peroxynitrite increase Arg-I gene expression in porcine coronary arterioles (Thengchaisri et al., 2006). An increase in Arg-II but not Arg-I expression has been suggested to play a role in women with preeclampsia, which is also mediated by peroxynitrite (Sankaralingam et al., 2010). Interestingly, in both cases, Rho-ROCK is the signaling mechanism involved in Arg-I in porcine and Arg-II in human endothelial cells (Thengchaisri et al., 2006; Chandra et al., 2012). The importance of Rho-ROCK pathway in the regulation of arginase gene expression and activity is also demonstrated by other studies including ours with the stimuli such as thrombin (Ming et al., 2004), oxidized LDL (Ryoo et al., 2011) and hyperglycemia (Romero et al., 2008; Toque et al., 2013). The fact that statins inhibit arginase activity in endothelial cells involving Rho/ROCK pathway could one of the mechanisms contributing to the beneficial effects of the drugs in treatment of cardiovascular disease (Ming et al., 2004; Holowatz et al., 2011).

In addition, p38MAPK (mitogen-activated protein kinase) is also implicated in regulation of arginase expression and activity in endothelial cells. p38MAPK is a member of the superfamily of MAPKs which serves as cellular a stress sensor for a variety of cellular stresses including hyperglycemia, oxidative stress, and inflammatory cytokines (Denise et al., 2012). It has been demonstrated that activation of the p38MAPK in macrophages increases arginase activity and expression of Arg-I (Stempin et al., 2004) and Arg-II (Liscovsky et al., 2009). This seems to be true in bovine and rat aortic endothelial cells for Arg-I expression (Zhu et al., 2010) and in human endothelial cells and mouse penile tissues for Arg-II expression in response to angiotensin-II (Toque et al., 2010) and persistent exposure to insulin (Giri et al., 2012). Moreover, *in vivo* treatment of hypertensive mouse induced by angiotensin-II infusion with a p38MAPK inhibitor prevents elevation of Arg-II expression and activity and enhances endothelium-dependent relaxation (Toque et al., 2010).

Most recently, we have demonstrated a crosstalk between S6K1 (40S ribosomal protein S6 Kinase-1) and Arg-II in endothelial cells (Yepuri et al., 2012), which are importantly involved in vascular aging. Our previous study showed that S6K1 activity is persistently high in senescent human endothelial cells and in the aortas of old rodents, which plays a causal role in age-associated eNOS-uncoupling and endothelial senescence (Rajapakse et al., 2011). Interestingly, overexpression of a constitutively active S6K1 mutant upregulates Arg-II (not Arg-I) gene expression and arginase activity in non-senescent cells by stabilizing Arg-II mRNA (Yepuri et al., 2012). Conversely, silencing S6K1 in senescent cells reduces Arg-II gene expression and activity and genetic or pharmacological inhibition of S6K1 in senescent cells or in old rat aortas decreases Arg-II gene expression and activity, demonstrating a critical role of hyperactive S6K1 in up-regulating Arg-II gene expression resulting in enhanced arginase activity in endothelial aging. Furthermore, our study also shows that silencing Arg-II gene in senescent endothelial cells inhibits S6K1 activity and Arg-II gene knockout in mouse abolishes age-associated hyperactive S6K1 in the aortas, demonstrating a feedforward cycle between S6K1 and Arg-II is present in vascular endothelial aging. Interruption of this crosstalk either by inhibition of S6K1 or Arg-II can recouple eNOS function, leading to reduced oxidative stress, improved NO^⋅^ production, inhibition of endothelial adhesion molecule expression, monocyte-endothelial cell interaction, and cell senescence markers in aging. Thus, the mutual positive regulation between S6K1 and Arg-II gene expression accelerates endothelial aging through eNOS-uncoupling, leading to oxidative stress and inflammation (Yepuri et al., 2012). The results suggest that interruption of S6K1-Arg-II crosstalk may represent a promising therapeutic strategy to decelerate vascular aging and age-associated cardiovascular diseases. Future work shall investigate the exact mechanisms how S6K1 stabilizes Arg-II mRNA, and how Arg-II activates S6K1 in the endothelial cells. The signaling mechanisms that regulate vascular arginase expression/activity are also summarized in the Figure 2.

## Perspectives of Targeting Arginase in Cardiovascular Diseases

Arginase-II as therapeutic target in cardiovascular diseases has shown promising beneficial effects in genetic modified mouse models. Systemic deficiency of Arg-II reduces systemic and vascular inflammations in mice fed high cholesterol diet and high fat diet, and improves endothelial function in aging, reduces atherosclerosis, and improves insulin sensitivity and glucose homeostasis (Ming et al., 2012; Yepuri et al., 2012). Conversely, endothelial specific Arg-II transgenic mice on ApoE^−*/*−^ background show accelerated atherosclerosis (Vaisman et al., 2012). Although some studies implicate that targeting Arg-I is also of therapeutic relevance in cardiovascular diseases, the firm evidence is lacking, which is due to the fact that systemic Arg-I deficient mouse exhibited severe symptoms of hyperammonemia, and died between postnatal days 10 and 14 (Iyer et al., 2002), and endothelial specific Arg-I knockout mouse is not available, yet, and the studies are solely dependent on the pharmacological inhibitors which inhibit both isoforms of arginases (The chemical characteristics and pharmacological effects of available arginase inhibitors are summarized in the Table 1). Nevertheless, the therapeutic potential of targeting arginases with these inhibitors has been proved in a number of experimental models of cardiovascular disease as discussed (Yang and Ming, 2006b; Pernow and Jung, 2013). Small scale human studies with local administration of arginase inhibitors investigating vascular endothelial functions as primary end point showed promising results in improving skin blood flow in elderly human subjects (Stanhewicz et al., 2012), in hypertensives (Holowatz and Kenney, 2007), and in patients with coronary artery disease and type 2 diabetes (Shemyakin et al., 2012). These inhibitors could theoretically inhibit liver Arg-I and may lead to hyperammonemia, although this side effect has not been reported in animals treated with arginase inhibitors for studies designed to investigate the role of arginase in vascular disease (Bagnost et al., 2010). It is clear that isoform-specific arginase inhibitors should be developed.

**Table 1:** **Available arginase inhibitors**.

Name (references)	Chemical class	Isoform-selectivity	Inhibitory mechanism
α-Difluoromethylornithine (DFMO) (Selamnia et al., 1998 )	L-Ornithine analog	Non-isoform-selective *K*_i_ = 3.9 ± 1.0 mM for arginase in HT-29 homogenate	Poor arginase inhibitor (commonly used as a specific ODC irreversible inhibitor)
L-Ornithine (*^*3*^*Reczkowski and Ash, 1994*^*1*^*; Colleluori and Ash, 2001; *^*2*^*Colleluori et al., 2001)		More potent in inhibiting hepatic arginase*^*1*^ K*_i_ = 1 mM for Arg-I*^*3*^ K*_i_ > 10 mM for hArg-II*^*2*^*	Competitive inhibition
L-Valine (Colleluori et al., 2001)	Branched-chain amino acid	Non-isoform-selective *K*_i_ = 0.4 mM for hArg-II	Non-competitive
L-Norvaline (Colleluori et al., 2001)	An analog of L-valine	*K*_i_ = 0.4 mM for hArg-II	Non-competitive
*N*^ω^-Hydroxy-L-arginine (NOHA) (Boucher et al., 1994; Buga et al., 1996; *^*5*^*Custot et al., 1997; *^*4*^*Baggio et al., 1999; Cox et al., 2001)	*N*^ω^-OH-based arginine analog	More potent in inhibiting hepatic arginase*^*4*^ K*_i_ = 10 μM for rArg-I*^*5*^ K*_i_ = 1.6 μM for hArg-II*^*1*^*	Competitive inhibitor. (an intermediate in NO synthesis, acts also as a substrate for the NOS)
*N*^ω^-Hydroxy-nor-L-arginine (nor-NOHA) (Custot et al., 1997)	*N*^ω^-OH-based arginine analog	*K*_i_ = 0.5 μM for rArg-I*^*5*^ K*_i_ = 51 nM for hArg-II*^*1*^*	Competitive inhibitor
*S*-(2-boronoethyl)-L-cysteine (BEC) (^6^Kim et al., 2001)	Boronic acid-based arginine analog	Non-isoform-selective. *K*_i_ = 0.4–0.6 μM for rArg-I*^*6*^ K*_i_ = 0.31 μM for hArg-II*^*1*^*	Competitive inhibitor
2(*S*)-amino-6-boronhexanoic acid (ABH) (^7^Baggio et al., 1999; ^8^Van Zandt et al., 2013)	Boronic acid-based arginine analog	More potent in inhibiting extrahepatic arginase*^*7*^*: *K*_i_ = 190 nM for hepatic Arg. *K*_i_ = 18–50 nM for extrahepatic non-isoform-selective*^*8*^*: *K*_i_ = 1.45 μM for hArg-I *K*_i_ = 1.92 μM for hArg-II	Competitive inhibitor
(R)-2-amino-6-borono-2-(2-(piperidin-1-yl)ethyl)hexanoic acid (compound 9) (Van Zandt et al., 2013)	Aminoethylene ABH analog (α,α-disubstituted amino acid-based)	Non-isoform-selective: *K*_i_ = 223 nM for hArg-I *K*_i_ = 509 nM for hArg-II	N/A

*K_i_, the inhibitor constant; ODC, ornithine decarboxylase; rArg-I, rat arginase-I; hArg-II, human arginase-II; N/A, not available. None of these inhibitors are really isoform-specific, although some of the inhibitors, such as nor-NOHA, have been reported to be more potent against one of the isoform in one study, but not in the other study. It is also to notice that the *K*_i_ is not always determined for both of the isoforms in the same study. In this case, the reference (the superscript number in italic) is given for the two K_i_*.

It is however, interesting to notice that pharmacological agents that target arginase indirectly through blockade of signaling transduction pathways that regulate arginase gene expression or activity show beneficial effect on vascular functions. Early studies demonstrate that statins which inhibit arginase activity through inhibition of the small G protein or GTPase RhoA improves endothelial function (Ming et al., 2004; Holowatz et al., 2011). Similarly, pharmacological and genetic inhibition of ROCK, the down-stream kinase of RhoA, showed similar inhibitory effects on arginase activity and endothelial dysfunction in atherosclerotic, diabetic, and angiotensin-II-induced hypertensive animal models (Ming et al., 2004; Shatanawi et al., 2011; Yao et al., 2013). Moreover, rapamycin and resveratrol which are capable of inhibiting mTORC1-S6K1 signaling pathway can also inhibit arginase activity and recouples eNOS function in aging animal models (Rajapakse et al., 2011; Yepuri et al., 2012). Furthermore, p38MAPK inhibitors have been shown to improve endothelial function also through inhibition of eNOS-uncoupling in endothelial cells or mouse aortas exposed to glucosamine (Wu et al., 2012) or inhibition of arginase in the corpora cavernosa from angiotensin-II-treated mice (Toque et al., 2010). Recently, a small clinical study has also showed that p38MAPK inhibitors improves endothelial function and reduces systemic and vascular inflammation in patients with hypercholesterolemia and coronary artery disease (Cheriyan et al., 2011; Elkhawad et al., 2012). With all these pharmacological inhibitors one can not precisely assess how much of the effects is really attributable to the inhibition of arginase activity. Depending on the functions of the signaling pathways in cardiovascular diseases, the off-target effects of these drugs could be of great therapeutic relevance in cardiovascular diseases.

## Conflict of Interest Statement

The authors declare that the research was conducted in the absence of any commercial or financial relationships that could be construed as a potential conflict of interest.
